# The metabolic reprogramming of γ-aminobutyrate in oral squamous cell carcinoma

**DOI:** 10.1186/s12903-024-04174-0

**Published:** 2024-04-05

**Authors:** Shi-Lian Wu, Guang-Yu Zha, Ke-Bin Tian, Jun Xu, Ming-Guo Cao

**Affiliations:** School of Medicine, Lishui University, No 01, Rd Xueyuan Avenue, Lishui, 323000 Zhejiang China

**Keywords:** Oral squamous cell carcinoma, Metabolic reprogramming, γ-aminobutyrate

## Abstract

Oral squamous cell carcinoma (OSCC) is the most common head and neck malignancy. The oncometabolites have been studied in OSCC, but the mechanism of metabolic reprogramming remains unclear. To identify the potential metabolic markers to distinguish malignant oral squamous cell carcinoma (OSCC) tissue from adjacent healthy tissue and study the mechanism of metabolic reprogramming in OSCC. We compared the metabolites between cancerous and paracancerous tissues of OSCC patients by ^1^HNMR analysis. We established OSCC derived cell lines and analyzed their difference of RNA expression by RNA sequencing. We investigated the metabolism of γ-aminobutyrate in OSCC derived cells by real time PCR and western blotting. Our data revealed that much more γ-aminobutyrate was produced in cancerous tissues of OSCC patients. The investigation based on OSCC derived cells showed that the increase of γ-aminobutyrate was promoted by the synthesis of glutamate beyond the mitochondria. In OSCC cancerous tissue derived cells, the glutamate was catalyzed to glutamine by glutamine synthetase (GLUL), and then the generated glutamine was metabolized to glutamate by glutaminase (GLS). Finally, the glutamate produced by glutamate-glutamine-glutamate cycle was converted to γ-aminobutyrate by glutamate decarboxylase 2 (GAD2). Our study is not only benefit for understanding the pathological mechanisms of OSCC, but also has application prospects for the diagnosis of OSCC.

**Supplementary Information:**

The online version contains supplementary material available at 10.1186/s12903-024-04174-0.

## Introduction

Oral squamous cell carcinoma (OSCC) is the most common head and neck malignancy, and its incidence has been increasing in several countries [1]. OSCC develops in the oral cavity and oropharynx and can occur due to many etiological factors [2]. Potentially malignant disorders transforming into OSCC includes leukoplakia, proliferative verrucous leukoplakia, erythroleukoplakia, erythroplakia, oral submucous fibrosis and oral lichen planus [2]. Gene mutations may cause cancer development in the pharynx and oral cavity; however, no specific gene has been identified in OSCCs [3]. Up-to-date, several molecular markers of prognosis have been studied in OSCC, but none entered in the clinical setting [4]. Because they are overexpressed in OSCC relative to normal mucosa, cytokeratins (CKs) are possibilities for diagnostic markers of OSCC [5]. The expression of CK5/14 and vimentin correlated with a poor prognosis of oral cancer patients [6]. The immunohistochemical detection of CK5/14 and ki-67 was normally performed for the examination of OSCC [7].

Increasing evidence reveals that metabolomics techniques, including nuclear magnetic resonance (NMR) spectroscopy and mass spectrometry (MS), can be used to explore the oncometabolites in OSCC. Plasma, serum, saliva, urine, tissue, derivative cell lines, and animal models have been used in OSCC metabolomics research. Shigeyama et al. analyzed volatile organic compound and found that 20 volatile metabolites in saliva of OSCC patients were significantly reduced compared with healthy people, suggesting that these metabolites may be involved in the pathogenesis of OSCC and should be considered as potential biomarkers [8]. The analysis based on the differential markers between OSCC and oral non neoplastic bone disease showed that nonanoic acid, glucose, galactose and cysteine cystine were potential plasma biomarkers [9]. Another study employing biofluids other than saliva found that the level of valine in OSCC patients decreased, while the abundance of choline and acetone increased [10]. The metabolic alterations involved in OSCC pathogenesis have also been assessed in OSCC-derivative cell lines, animal models and tissues, showing that the metabolic signatures about glycolytic metabolic pathways [11], fatty acid metabolism [12] and other metabolic pathways [13] were characterized in OSCC.

However, the metabolic alterations, especially the mechanism of metabolic reprogramming [14], are not clearly elucidated in OSCC. To study the metabolic reprogramming characters of OSCC, we compared the metabolites between cancerous and paracancerous tissues of OSCC patients by ^1^HNMR analysis. We established OSCC derived cell lines and preliminarily evaluated the metabolic reprogramming of γ-aminobutyrate in OSCC. Our study is not only benefit for understanding the pathological mechanisms of OSCC, but also has application prospects for the diagnosis of OSCC.

## Materials and methods

### Patient eligibility

This study included 4 oral squamous cell carcinoma (OSCC) patients (patient ID: #21-3801, #21-5479, #21-6812 and #21-7123, each in Stage I/II) treated at The First Hospital of Jiaxing. Patients meeting all the following requirements were eligible for enrollment: (i) a diagnosis of OSCC confirmed by histology, (ii) no treatment before diagnosis, and (iii) provision of voluntary written informed consent. Human specimens included tumor tissue and adjacent normal tissue (beyond tumor margins) for analysis of the expression of the CK-5 and Ki-67. This study was carried out in accordance with the current guidelines of Medical Ethics Committee at the Medical Center of Lishui University (LSUMC-21-053).

### Cell lines establishing

The tumor and non-tumorous adjacent tissues were collected from one OSCC patient (#21-3801) who received surgical treatment at The First Hospital of Jiaxing and individually dissected into smaller fragments. The fragments from tumor or non-tumorous adjacent tissues were then seeded onto 10 cm plate and cultured in Dulbecco’s Modified Eagle’s Medium (DMEM) (Hyclone, SH30019.01, logan, UT) with 10% Fetal Bovine Serum (FBS). After the cells that migrated from the tissue block were connected into pieces, fibroblasts were manually scraped off under microscope. The left epithelial cells were cultured and passaged for three generations. The expanded cells were then applied to establish single cell clones by limiting dilution with 96 well plate.

### H&E staining

H&E staining was performed as previously described [15]. Briefly, 10-µm-thick cryosections were first incubated in Haematoxylin A solution for 3 min. After washing with water three times, the sections were rinsed in a concentrated HCl solution diluted with 70% ethanol for one minute and washed again with water three times. Then, the sections were incubated in 1% ammonia water for 1 min, washed for 3 times, stained with Eosin-Y solution for 8–10 s, dehydrated in a series of ethanol and xylene and mounted with neutral balsam. Olympus microscope (Olympus, DP72) was applied for images acquiring.

### Immunohistochemistry

Immunohistochemistry (IHC) was performed as previously described [16]. Briefly, the sample was fixed in 4% PFA at 4 °C for 1 h and washed 3 times with PBS. Then, the tissue was placed overnight in a 30% sucrose/PBS solution at 4 ° C. Afterwards, the samples were embedded in OCT compound (Sakura) and sliced. Seal the sections with PBSST (0.1% Triton X-100/2.5% normal donkey serum/PBS) at room temperature for 30 min, and then incubate them overnight with primary antibodies at 4 °C. Wash with PBS three times and incubate with the second antibody at room temperature for 30 min. Wash the slices three times with PBS and install them with media containing DAPI.

### Plasmids and siRNAs

Human tagged ORF clone for GLUL (#RC204161), ALDH2 (#RC200505), BLVRA (#RC203243) and FBP1 (#RC204461), and human siRNA oligo duplex for GLUL (#SR301833), ALDH2 (#SR319314), BLVRA (#SR300442), FBP1 (#SR301541), GLS(#SR301829) and GAD2(#SR301713) were purchased from OriGene Technologies. To make overexpression cells, GLUL, ALDH2, BLVRA or FBP1 cDNA plasmids were transfected into indicated cells and selected by Neomycin.

### ^*1*^H-NMR measurements

The tissue samples were thawed and meshed at room temperature and centrifuged at 8000 rpm for 5 min. 150 µL of supernatant was added to 800 µL of methanol, mixed by vigorous vortex, and then centrifuged at 14 000 g for 10 min. The supernatant was concentrated by SpeedVac system at 35 C. The residue was redissolved in 450 µL of distilled water, 50.0 µL of phosphate buffer solution (pH 7.4), and 50.0 µL of TSP (0.5 mg/ml). After centrifugation with 12 000 rpm for 10 min, 500 µl supernatant was transferred to the 5 mm NMR tube and stored at 4 ℃ for analysis. ^1^H-NMR data of samples were collected on Bruker Avance II-600 MHz spectrometer (Germany), and the methods were consistent with those reported in literatures [17]. Topspin 2.1 (Bruker, Biospin, GmbH, Rheinstetten, Germany) was used for phase adjustment and baseline correction of each 1H NMR spectrum, and the TSP chemical shift was calibrated to 0.00 ppm. The processed ^1^H NMR spectra were integrated into Matlab-R2012b software (Mathworks, Natick, MA, USA) to cover the entire spectrum from 0 to 10.0 ppm. For eliminating the influence of peaks of water and methanol, the data of 3.36 to 3.37 and 4.5 to 5.5 ppm region were deleted.

### RNA extraction and quantitative real-time PCR

RNA extraction and quantitative Real-time PCR were performed as previously described [18]. Firstly, use TRIzol ® The reagent (Invitrogen; Thermo Fisher Scientific, Inc.) isolated total RNA from the corresponding cell samples. Reverse transcription of RNA into cDNA using PrimeScript RT kit (Takara Biotechnology Co., Ltd.). Using cDNA as a template, SYBR Premix Ex Taq ™ II. PCR forward primer (10 µ M), Polymerase chain reaction reverse primer (10 µ M) Using ROX reference dye and sterile distilled water as raw materials, PCR amplification was performed under the following conditions: 95 ˚C pre denaturation for 10 min; 40 cycles of denaturation at 95˚C for 10 s, at 60˚C for 20 s.

### RNA sequencing and data analysis

RNA sequencing and data analysis were performed as previously described [15]. Briefly, RNAs from CA-13 and PA-35 cells were used for RNA-seq. Total RNA was extracted using the TRIzol reagent (Invitrogen) and identified Agilent 2100 bioanalyzer (Thermo Fisher Scientific, Waltham, MA, USA). Subsequently, rRNA was removed from the identified RNA using the Ribo Zero Magnetic Kit (Epicentre). After constructing the RNA library using the Epi™ mini long RNA-seq kit, the Illumina NovaSeq 6000 instrument was used for sequencing. The sequencing data was first analyzed for significance and false discovery rate (FDR), and then the DESeq2 algorithm is applied to filter differentially expressed genes.

### Metabolites measurement

Metabolites from indicated cells were measured by colorimetric assay sit for glutamine (#E-BC-K853-M, Elabscience Technologies, Inc.) and GAB (#E-BC-K852-M, Elabscience Technologies, Inc.) following the instruction of the manufacturers. Briefly, the culture medium of cells was removed and washed with PBS (0.01 M, pH 7.4), and then collected by trypsin treatment. Cells were then resuspended in 2–5 mL PBS (0.01 M, pH 7.4) for test by ELx800 Enzyme marker (Shanghai Ranger Apparatus Co.,Ltd.).

The production of metabolites was quantified by BioTek Gen5™ at wavelength 640 nm (GAB) or 450 nm (Glutamine).

### Western blotting

Cells were harvested and applicated in the isolation of enriched mitochondrial proteins (MITOISO2 kit, Sigma-Aldrich). The mitochondrial fraction and remained proteins were individually dissolved in lysis buffer containing Complete Protease Inhibitor Cocktail (Roche) and subjected to SDS-PAGE. Cellular proteins were transferred onto a nitrocellulose membrane (Millipore) for anti-GLUL (#GT1055, Thermo Fisher Scientific.; dilution 1:1000), anti-GLS (#701,965, Thermo Fisher Scientific.; dilution 1:1000), anti-GAD1 (#ab237998, Abcam.; dilution 1:2000), anti-GAD2 (#CSB-PA11159B0Rb, CUSA Biotechnology.; dilution 1:500) or anti-GAPDH (#9484, Abcam.; dilution 1:5000) antibodies. After washing, the membranes were incubated with secondary antibodies. The reaction was revealed using an ECL chemiluminescence kit (Amersham Biosciences) with Eastman Kodak Co. hyperfilm and quantified by Multi Gauge software (Fujifilm Life Sciences). Data are presented as the mean ± SD, based on at least three independent repeats.

### Statistics

All data were determined from 5 independent experiments and are presented as mean values + SD. Differences between groups were analyzed using a Student’s *t*-test. The differences were deemed statistically significant at *P* < 0.05.

## Results

### Identification of metabolites for the OSCC patients

**Fig. 1:**
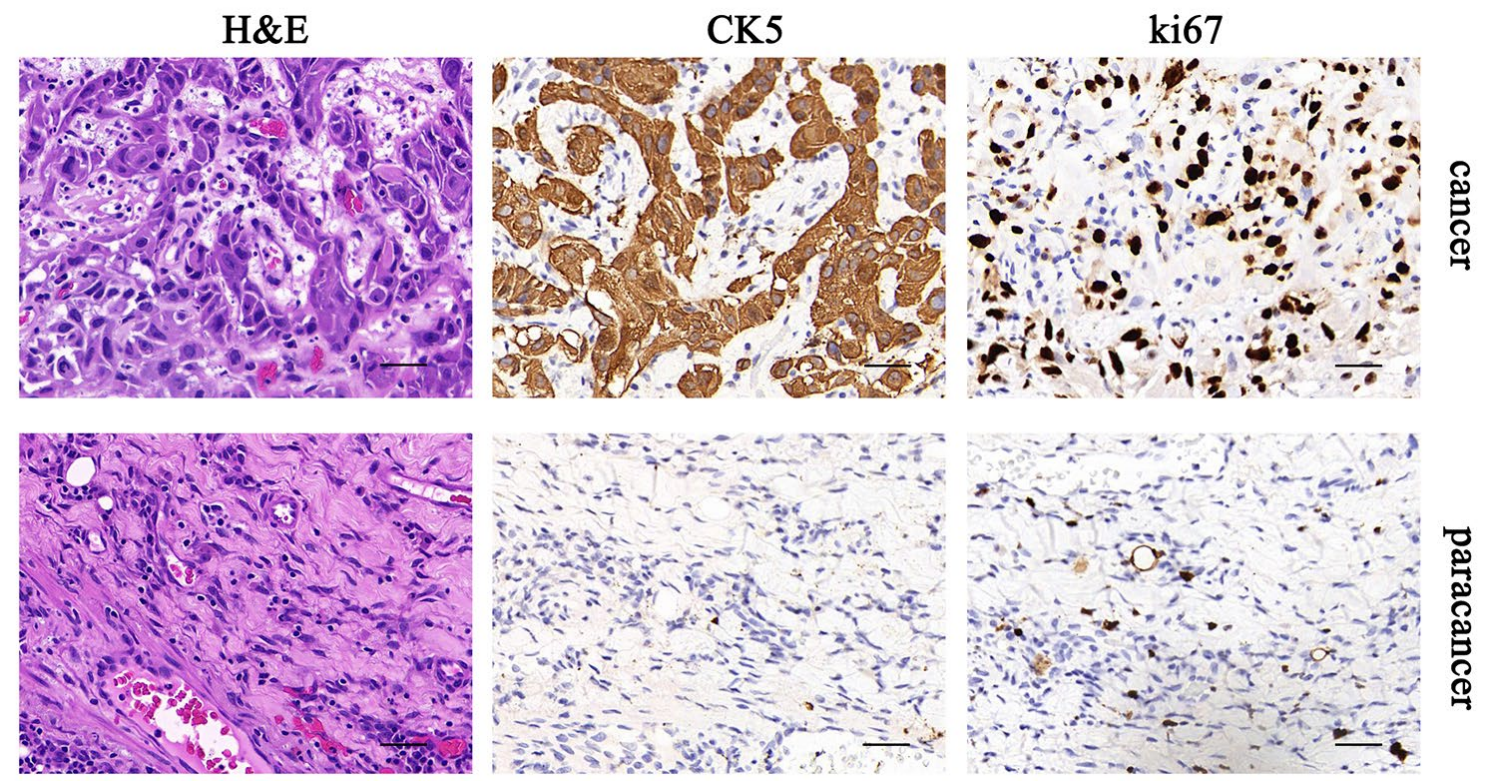
Pathological examination of OSCC patients. The masses of patients with oral squamous cell carcinoma removed surgically were sent for pathological examination. Representative images showed the detection of H&E and immunohistochemistry (Ki67 and CK5). Scale bar, 20 μm

**Fig. 2:**
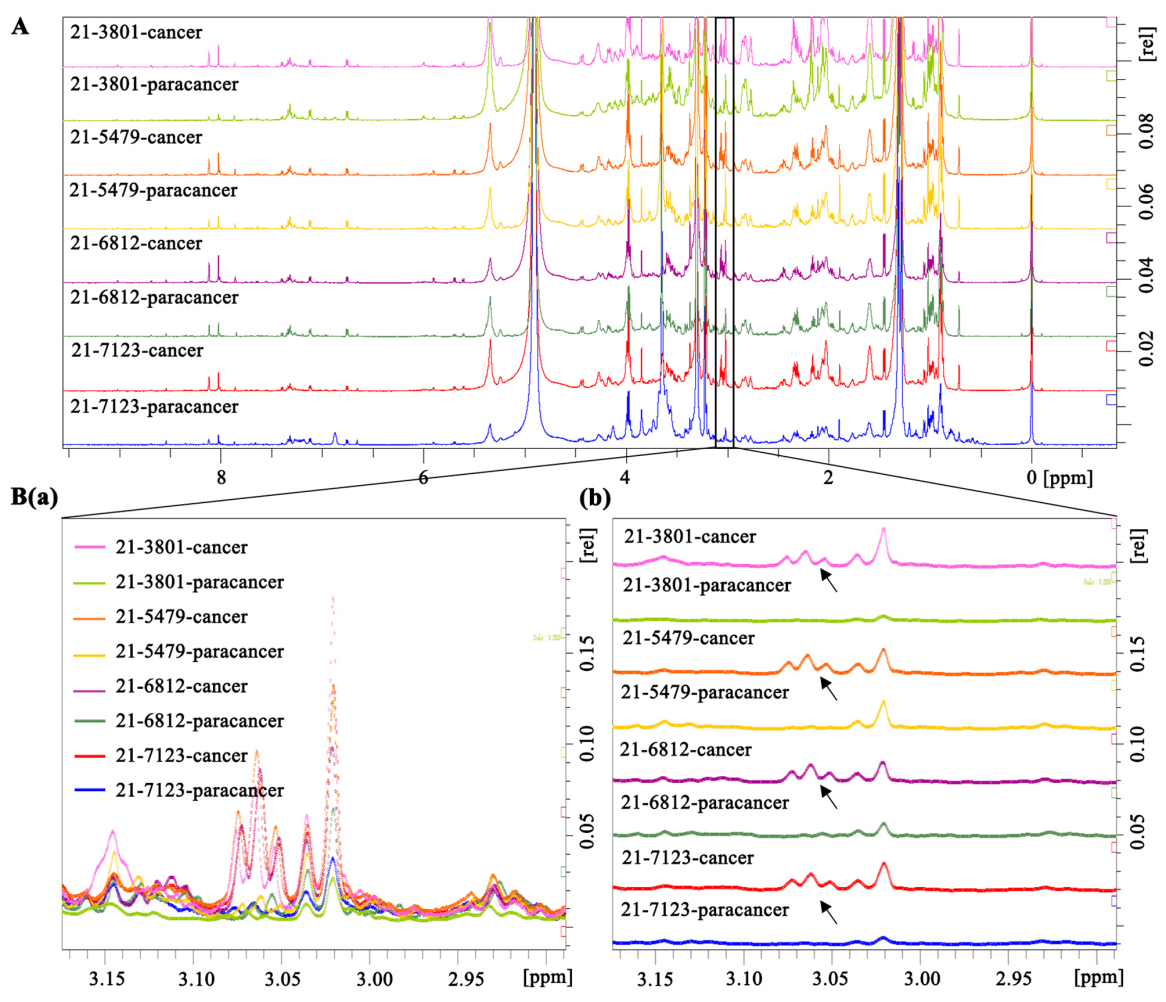
Analysis of metabolites in cancer and paracancerous tissues of OSCC patients. **(A)** The metabolites in cancer and paracancerous tissues of 4 OSCC patients (#21-3801, #21-5479, #21-6812 and #21-7123) were analyzed by ^1^H-NMR. **(B)** The signal in the range of 2.9–3.2 ppm was selected to export the original data. The layout was toggled as merged (a) or multiple displayed (b). The arrows show the sites of metabolite with density variation in cancer and paracancerous tissues

To investigate the metabolic characteristics of oral squamous cell carcinoma (OSCC), we compared the metabolites in the cancerous and paracancerous tissues (Fig. 1) of 4 patients with OSCC. The ^1^HNMR analysis showed that the density of metabolites was varied among different tissues detecting by relative chemical shift between 0 and 10 ppm. While some metabolites were highly expressed in cancerous tissues (Fig. 2A and Supplementary Fig. 1C), others showed much lower expression or were hardly detectable (Supplementary Fig. 1A), indicating that the procedure of metabolic reprogramming had been activated in cancer tissue. In particular, the ^1^HNMR spectrum showed that the metabolites with relative chemical shifts between 3.04 and 3.08 ppm was promoted in cancer tissues (Fig. 2B). Through literature review [17] and comparison of standard product library, the substance with three consecutive peaks in the ^1^HNMR spectrum was identified as γ-aminobutyrate (GAB).

### Transcriptional profiling of OSCC patient derived cells

**Fig. 3:**
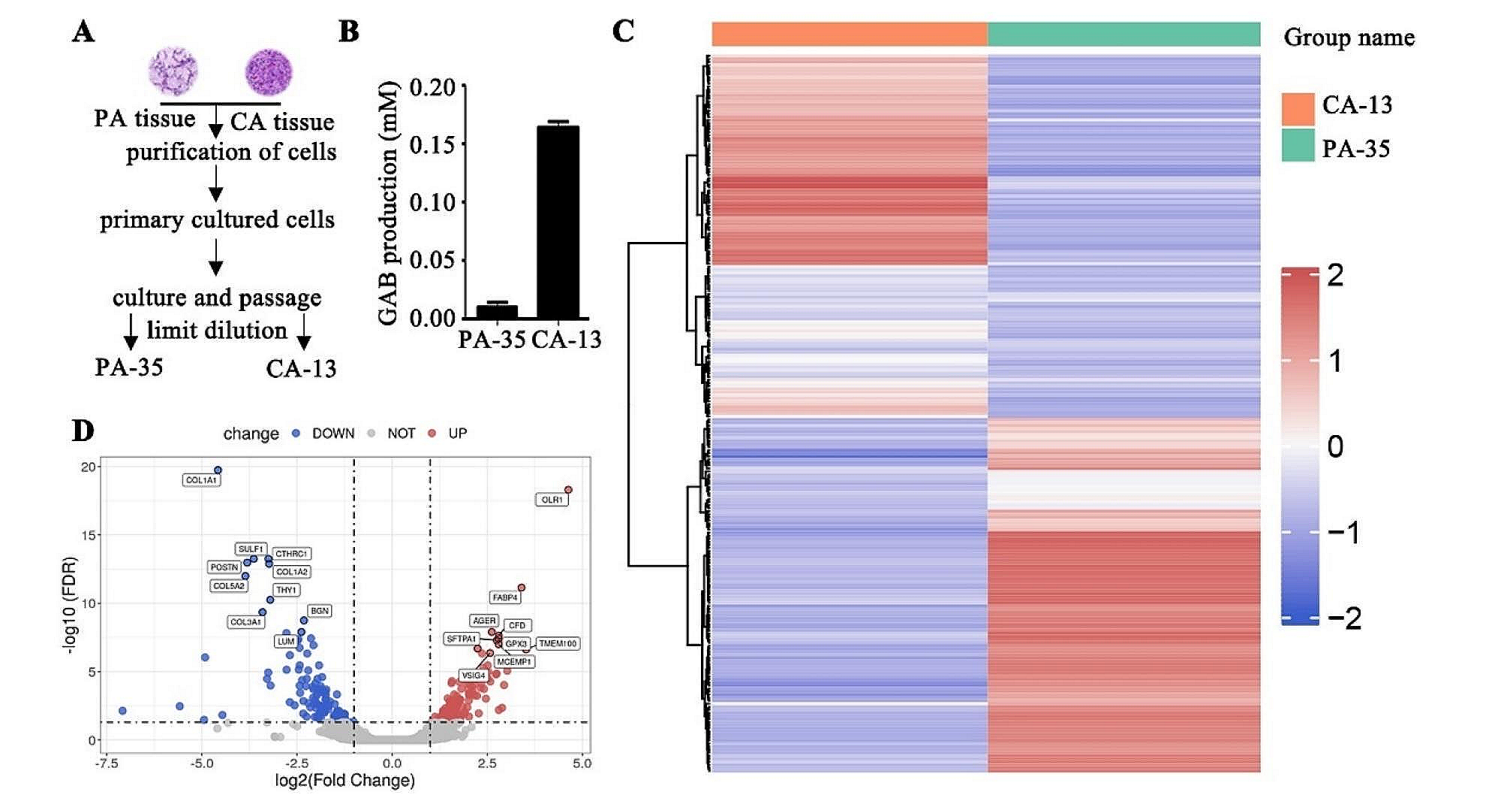
Transcriptional profiling of OSCC patient derived cells. **(A)** The scheme for establishing OSCC patient derived CA-13 and PA-35 cells (CA, cancer; PA, paracancer). **(B)** GAB quantities of CA-13 and PA-35 cells were determined by Colorimetric Assay Kit. **C and D.** The cluster analysis was developed on the differential genes between CA-13 and PA-35 cells. The thresholds used were FDR < 0.05 and the absolute value of log2 (Fold Change) > 1, respectively. The results were showed as heat map (B) or volcano map (**C**)

To validate the features of high GAB yield in the samples of cancer tissue, we established a series of cell lines from cancerous or paracancerous tissues of one OSCC patient (Fig. 3A). The production of GAB was evaluated in cancer tissue derived CA-13 cells and paracancerous tissue derived PA-35 cells. In consistent with the clinical samples, much more endogenous GABs were detected in CA-13 cells than in PA-35cells (Fig. 3B), suggesting that the metabolism of GAB was differently regulated between cancerous and paracancerous tissues.

To better understand the regulation mechanism of GAB metabolism, we next employed RNA sequencing to examine the RNA expression of cancer tissue derived CA-13 cells and paracancerous tissue derived PA-35 cells. Comparing with PA-35 cells, RNA-Seq revealed that CA-13 cells had a total of 234 genes with significant differential expression, including 115 up-regulated genes and 119 down regulated genes (Fig. 3C and D, Data file 1). Among these differential genes, some were involved into neutrophil mediated immune function, and some were associated with extracellular matrix organization or external encapsulating structure organization (Supplementary Fig. 2).

### Involvement of GLUL into GAB metabolism of OSCC

**Fig. 4:**
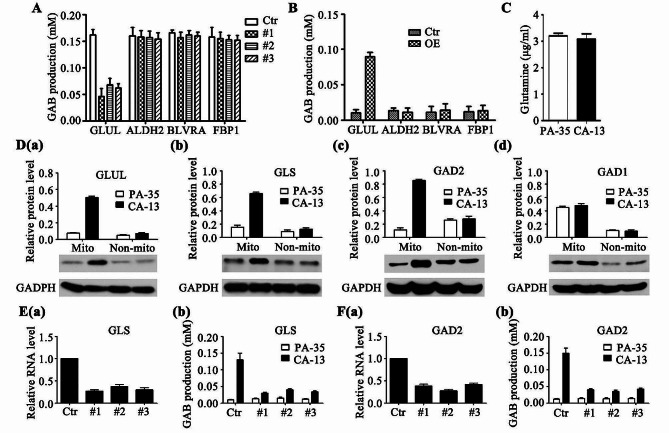
Involvement of GLUL into GAB metabolism

GAB was a metabolite of glutamate [19, 20] and much more endogenous GAB were contained in cancer tissue derived CA-13 cells than in paracancer tissue derived PA-35cells (Fig. 3B). Thus, to further figure out the critical genes involved into GAB metabolism of OSCC, we checked the KEGG enrich genes list (Data file 2) and focused on the candidate genes related to amino acid metabolism. Several genes relating to alanine, aspartate, glutamate, histidine, tryptophan, porphyrin, arginine, and proline metabolism were next tested for their influence on GAB production. The siRNA oligos for GLUL, ALDH2, BLVRA or FBP1 were introduced into cancer tissue derived CA-13 cells (Supplementary Fig. 3A). The results showed that the quantities of GAB were hardly changed between ALDH2, BLVRA or FBP1 knocking down CA-13 cells and their control cells (Fig. 4A). However, the GAB level in GLUL siRNA transfecting CA-13 cells was significantly inhibited (Fig. 4A). Moreover, the overexpression of GLUL in PA-35 cells promoted the yields of GAB, which were not varied with the increase of ALDH2, BLVRA or FBP1 (Fig. 4B and Supplementary Fig. 3B). These data demonstrated that GLUL, rather than ALDH2, BLVRA or FBP1, was involved into the metabolism of GAB in OSCC cells.

GLUL, encoded glutamine synthetase, is an enzyme that converts glutamate and ammonia to glutamine [21]. We measured the quantities of glutamine in CA-13 and PA-35 cells and found that they are no apparent distinction (Fig. 4C), indicating that the GLUL-converted-glutamine was consumed by other pathway. The metabolism of glutamine in cancer are overall highly heterogeneous that varies with a range of parameters, including the tissue of origin of a cancer, the genetic aberrations which drive it, the tumor microenvironment, and possibly diet and host metabolism [22]. Since the glutaminase (GLS), which catalyze glutamine to glutamate, was listed in KEGG enrich up-regulated genes (Data file 2), we compared the RNA level of GLS between CA-13 and PA-35 cells. The results confirmed that the expression of GLS was remarkably induced in CA-13 cells (Supplementary Fig. 3C), suggesting that the glutamine produced from GLUL catalyzing was consumed by GLS mediated glutamine to glutamate conversion.

### The alternative GAB metabolism in OSCC

In CA-13 cell, glutamate was converted to glutamine by GLUL, and then the generated glutamine was metabolized to glutamate by GLS. This glutamate-glutamine-glutamate cycle suggests that GAB, as the product of glutamate, may have alternative metabolic pathways in OSCC cells. Warburg effect suggest that defects in mitochondrial respiration may be the underlying cause of cancer [23], we thus speculate that the alternative metabolic pathway of GAB in OSCC is associated with the regulation of mitochondrial proteins. The enriched mitochondrial proteins were isolated and detected for the protein expression of GLUL, GLS and several genes related to GAB metabolism. Our results showed that GLUL and GLS were mainly up-regulated at non-mitochondrial sites in CA-13 cells, comparing with PA-35 cells. These data demonstrate that in CA-13 cells, the glutamate-glutamine-glutamate cycle occurs outside the mitochondria.

**Fig. 5:**
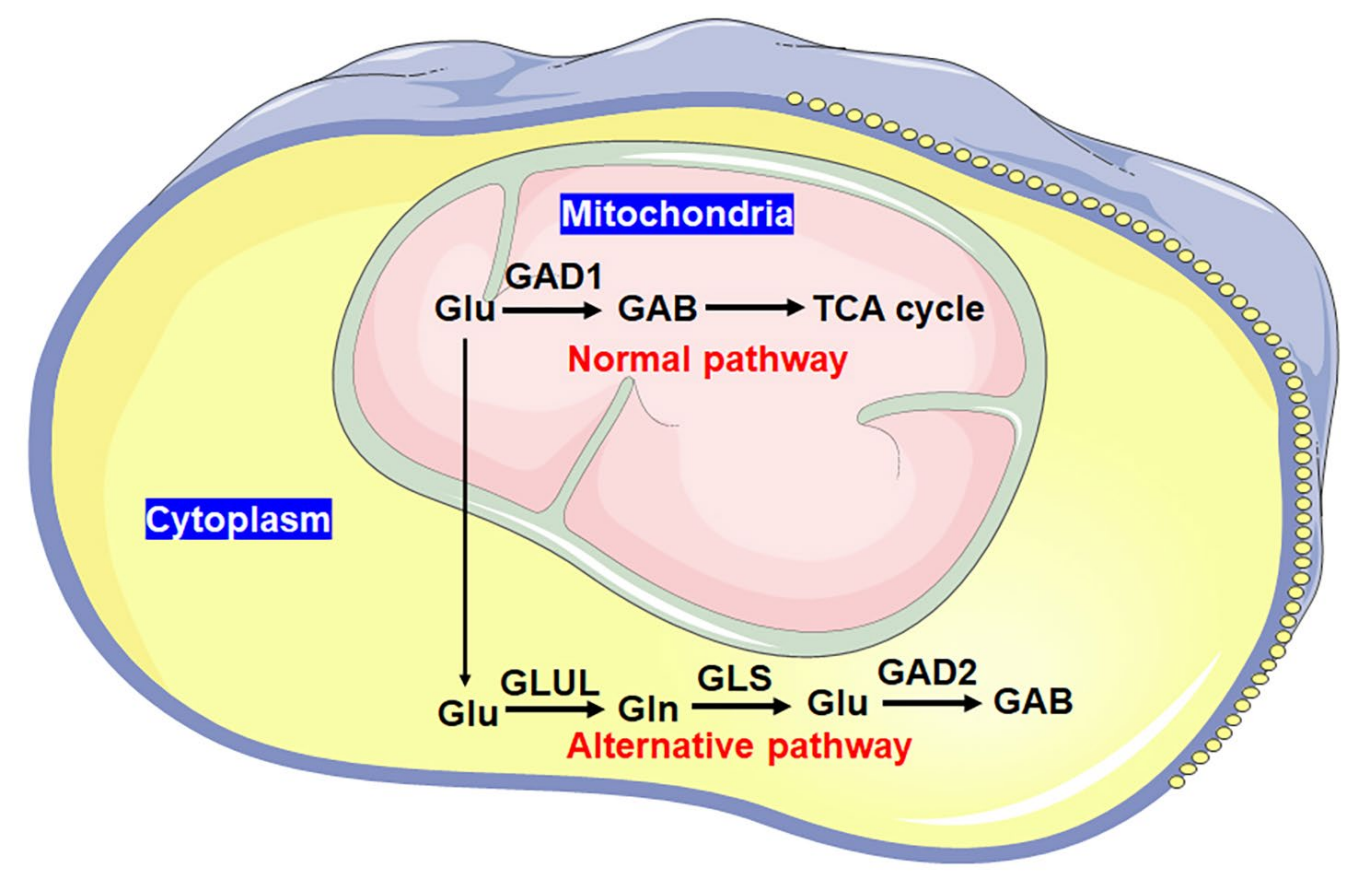
The diagram of GAB metabolism. Normally, the glutamate is catalyzed by GAD1 to GAB and then metabolized into TCA cycle in mitochondria. In OSCC cells, the glutamate is transpoted out of mitochondria and metabolized to GAB according the alternative pathway

Then we ask how the glutamate produced by the glutamate-glutamine-glutamate cycle is converted into GAB in the alternative metabolic pathway of GAB. Since two types of glutamate decarboxylase (GAD) were listed in 234 candidate genes (Data file 1), we measured the spatial expression of GAD1 and GAD2 in PA-35 or CA-13 cells. Compared with PA-35 cells, the expression of GAD1 in mitochondria of CA-13 cells was slightly inhibited, while that of GAD2 in non-mitochondrial sites was promoted (Fig. 4D), proving that the glutamate produced by the glutamate-glutamine-glutamate cycle was consumed by GAD2 in CA-13 cells. Moreover, we introduced GLS and GAD2 siRNA oligos into PA-35 or CA-13 cells and found that the blockage of GLS and GAD2 were both able to inhibit the production of GAB in CA-13 cells instead of PA-35 cells (Fig. 4E and F). Together, these data reveal that in OSCC, GAB was produced from an alternative pathway of glutamate metabolism mediated by GLUL, GLS and GAD2 (Fig. 5).

## Discussion

The molecular pathogenesis of OSCC is very complex, which is the result of several interdependent molecular mechanisms, involving not only changes in the expression of certain genes and proteins, but also the altered production of metabolites in metabolic processes. The biomarker research of OSCC tissue samples mainly focuses on identifying potential metabolic markers to distinguish malignant tumor tissue from adjacent healthy tissue [24]. Albeit the use of metabolomics in investigations aiming to identify novel OSCC biomarkers is still at an initial stage, the validation of individual and/or multiple groups of metabolites is strongly encouraged. Here, we reported that the production of GAB was elevated in OSCC cancer tissue and cancer tissue derived cells, indicating that the hyper-productivity of GAB could be one of the metabolic signatures of OSCC. Further verification and confirmation on more clinical samples are needed to determine whether the production of GAB alone can be used as a metabolic biomarker. However, the amount of GAB can be possibly developed as the diagnostic supplement biomarker for OSCC in addition to immunohistochemical examination on the expression of OSCC-associated proteins. Considering that histopathological evaluation is not sufficient to correctly define a healthy margin, metabolic markers may help ensure complete tumor removal, since unexpected local recurrence may occur due to cancer-associated genetic or epigenetic alterations in microscopically “normal epithelial cells’’ [25]. In this regard, GAB can also be developed to distinguish the healthy margin before surgery. We can confirm the surgical boundary by comparing the production of GAB in samples from different areas around the tumor lesion of OSCC patients using effective metabolites collection tool.

The increase in GAB production can be attributed to the active metabolism of glutamate, but the glutamate here comes from GLUL-catalyzed glutamine. Cancer cells are damaged in mitochondrial respiratory function, which leads to their preferentially glycolysis through Warburg effect even under sufficient oxygen [26]. Based on Warburg effect in cancer cells, we speculate that there is an alternative pathway for GAB metabolism in OSCC beyond mitochondria. Normally, glutamate is metabolized to GAB, and GAB is converted to succinic acid and enters the tricarboxylic acid (TCA) cycle [27]. In cancer cells, the TCA cycle is inhibited, and the GAD1-catalysed GAB inside mitochondria is accumulated, owing to the reprogramming of glucose metabolism. The blockage of glutamate-GAB metabolic pathway is fed back to the metabolism of glutamate. Thus, the glutamate is transported out of mitochondria to generate GAB in an alternative way (Fig. 5).

GAB, the biochemical form of γ-aminobutyric acid (GABA), participates in shaping physiological processes, including the immune response of T cells [20], modulating synaptic transmission, promoting neuronal development and relaxation, and preventing sleeplessness and depression [28]. Notably, plants might shift the cyclic flux from the TCA cycle to an alternative noncyclic pathway via GABA shunt under specific physiological conditions [29]. Although the mitochondria retain all required enzymes for an intact TCA cycle, our findings suggest that in OSCC, when the TCA cycle on mitochondria fails to proceed, glutamate metabolism switch to an alternative pathway. Whether this shift occurs actively or passively, its purpose is to adapt to the needs of environmental changes during the development of OSCC.

The alternative metabolism of GAB reflects the metabolic reprogramming of GAB in OSCC. Metabolic reprogramming is one of the important mechanisms for the occurrence and development of malignant tumors. Intervention of metabolic reprogramming is expected to become a new strategy for cancer prevention and treatment [14]. Although the metabolomics of OSCC has been carried out, most investigations were based on salivary or biofluid samples to identify panels of markers to discriminate OSCC from healthy controls, especially in early-stage tumors to improve early detection and prognosis. The special investigation for the mechanism of metabolic reprogramming of OSCC are still absent.

The amino acid glutamate is a major metabolic hub in many organisms [30]. Although we have made a preliminary study on the alternative mechanism of glutamate metabolism to GAB, there are still many questions to be answered. For example, what proteins or biomolecules are critical for transporting glutamate from mitochondria? Is there glutamate shuttle phenomenon between mitochondria and cytoplasm? Which signaling pathways are involved in the alternative mechanism of GLUT-regulated GAB metabolism? Further clarification will not only help to understand the mechanism of metabolic reprogramming in OSCC, but also play a positive role in the diagnosis and treatment of OSCC.

### Electronic supplementary material

Below is the link to the electronic supplementary material.

Supplementary Material 1

Supplementary Material 2

Supplementary Material 3

Supplementary Material 4

Supplementary Material 5

Supplementary Material 6

Supplementary Material 7

Supplementary Material 8

## Data Availability

The RNA-seq datasets generated for this study can be found in the Gene Expression Omnibus under accession GSE222879. The authors declare that all other data supporting the findings of this study are available within the paper and supplementary information files.

## References

[1] Du M, Nair R, Jamieson L, Liu Z, Bi P (2020). Incidence trends of lip, oral cavity, and pharyngeal cancers: global burden of Disease 1990–2017. J Dent Res.

[2] Bugshan A, Farooq I (2020). Oral squamous cell carcinoma: metastasis, potentially associated malignant disorders, etiology and recent advancements in diagnosis. F1000Res.

[3] Krishna A, Singh S, Kumar V, Pal US (2015). Molecular concept in human oral cancer. Natl J Maxillofac Surg.

[4] Russo D, Merolla F, Mascolo M, Ilardi G, Romano S, Varricchio S, Napolitano V, Celetti A, Postiglione L, Di Lorenzo PP et al. FKBP51 immunohistochemical expression: a New Prognostic Biomarker for OSCC? Int J Mol Sci 2017, 18(2). 10.3390/ijms18020443 PMC5343977 28218707

[5] Vaidya M, Dmello C, Mogre S. Utility of Keratins as biomarkers for human oral precancer and Cancer. Life (Basel) 2022, 12(3). 10.3390/life12030343 PMC8950203 35330094

[6] Mogre S, Makani V, Pradhan S, Devre P, More S, Vaidya M, Dmello C. Biomarker potential of Vimentin in oral cancers. Life (Basel) 2022, 12(2). 10.3390/life12020150 PMC8879320 35207438

[7] Kiyosue T, Kawano S, Matsubara R, Goto Y, Hirano M, Jinno T, Toyoshima T, Kitamura R, Oobu K, Nakamura S (2013). Immunohistochemical location of the p75 neurotrophin receptor (p75NTR) in oral leukoplakia and oral squamous cell carcinoma. Int J Clin Oncol.

[8] Shigeyama H, Wang T, Ichinose M, Ansai T, Lee SW (2019). Identification of volatile metabolites in human saliva from patients with oral squamous cell carcinoma via zeolite-based thin-film microextraction coupled with GC-MS. J Chromatogr B Analyt Technol Biomed Life Sci.

[9] Enomoto Y, Kimoto A, Suzuki H, Nishiumi S, Yoshida M, Komori T (2018). Exploring a novel screening method for patients with oral squamous cell carcinoma: a plasma Metabolomics Analysis. Kobe J Med Sci.

[10] Gupta A, Gupta S, Mahdi AA (2015). (1)H NMR-derived serum metabolomics of leukoplakia and squamous cell carcinoma. Clin Chim Acta.

[11] Knobloch TJ, Ryan NM, Bruschweiler-Li L, Wang C, Bernier MC, Somogyi A, Yan PS, Cooperstone JL, Mo X, Bruschweiler RP et al. Metabolic regulation of Glycolysis and AMP activated protein kinase pathways during black raspberry-mediated oral Cancer Chemoprevention. Metabolites 2019, 9(7). 10.3390/metabo9070140 PMC6680978 31336728

[12] Sant’Anna-Silva ACB, Santos GC, Campos SPC, Oliveira Gomes AM, Perez-Valencia JA, Rumjanek FD (2018). Metabolic Profile of oral squamous carcinoma cell lines relies on a higher demand of lipid metabolism in metastatic cells. Front Oncol.

[13] Vitorio JG, Duarte-Andrade FF, Dos Santos Fontes Pereira T, Fonseca FP, Amorim LSD, Martins-Chaves RR, Gomes CC, Canuto GAB, Gomez RS (2020). Metabolic landscape of oral squamous cell carcinoma. Metabolomics.

[14] Faubert B, Solmonson A, DeBerardinis RJ. Metabolic reprogramming and cancer progression. Science 2020, 368(6487). 10.1126/science.aaw5473 PMC7227780 32273439

[15] Xu JB, Cao J, Xia J, Zhu Y, He Y, Cao MG, Fang BM, Thiery JP, Zhou W (2023). Breast metastatic tumors in lung can be substituted by lung-derived malignant cells transformed by alternative splicing H19 lncRNA. Breast Cancer Res.

[16] Zhou W, Ye XL, Xu J, Cao MG, Fang ZY, Li LY, Guan GH, Liu Q, Qian YH, Xie D. The lncRNA H19 mediates breast cancer cell plasticity during EMT and MET plasticity by differentially sponging miR-200b/c and let-7b. Sci Signal 2017, 10(483). 10.1126/scisignal.aak9557 28611183

[17] Chen F, Zhang J, Song X, Yang J, Li H, Tang H, Liao YC (2011). Combined metabonomic and quantitative real-time PCR analyses reveal systems metabolic changes of Fusarium graminearum induced by Tri5 gene deletion. J Proteome Res.

[18] Cao M, Tian K, Sun W, Xu J, Tang Y, Wu S (2022). MicroRNA-141-3p inhibits the progression of oral squamous cell carcinoma via targeting PBX1 through the JAK2/STAT3 pathway. Exp Ther Med.

[19] He W, Wu G (2020). Metabolism of amino acids in the brain and their roles in regulating Food Intake. Adv Exp Med Biol.

[20] Kang S, Liu L, Wang T, Cannon M, Lin P, Fan TW, Scott DA, Wu HJ, Lane AN, Wang R (2022). GAB functions as a bioenergetic and signalling gatekeeper to control T cell inflammation. Nat Metab.

[21] Eelen G, Dubois C, Cantelmo AR, Goveia J, Bruning U, DeRan M, Jarugumilli G, van Rijssel J, Saladino G, Comitani F (2018). Role of glutamine synthetase in angiogenesis beyond glutamine synthesis. Nature.

[22] Cluntun AA, Lukey MJ, Cerione RA, Locasale JW (2017). Glutamine metabolism in Cancer: understanding the heterogeneity. Trends Cancer.

[23] Zong WX, Rabinowitz JD, White E (2016). Mitochondria and Cancer. Mol Cell.

[24] Hsu CW, Chen YT, Hsieh YJ, Chang KP, Hsueh PC, Chen TW, Yu JS, Chang YS, Li L, Wu CC (2019). Integrated analyses utilizing metabolomics and transcriptomics reveal perturbation of the polyamine pathway in oral cavity squamous cell carcinoma. Anal Chim Acta.

[25] Braakhuis BJ, Tabor MP, Kummer JA, Leemans CR, Brakenhoff RH (2003). A genetic explanation of Slaughter’s concept of field cancerization: evidence and clinical implications. Cancer Res.

[26] Martinez-Reyes I, Chandel NS (2021). Cancer metabolism: looking forward. Nat Rev Cancer.

[27] Li Y, Li YC, Liu XT, Zhang L, Chen YH, Zhao Q, Gao W, Liu B, Yang H, Li P (2022). Blockage of citrate export prevents TCA cycle fragmentation via Irg1 inactivation. Cell Rep.

[28] Ngo DH, Vo TS. An updated review on Pharmaceutical properties of Gamma-Aminobutyric Acid. Molecules 2019, 24(15). 10.3390/molecules24152678 PMC6696076 31344785

[29] Nehela Y, Killiny N (2022). Not just a cycle: three gab genes enable the non-cyclic flux toward Succinate via GABA Shunt in ‘Candidatus Liberibacter asiaticus’-Infected Citrus. Mol Plant Microbe Interact.

[30] Walker MC, van der Donk WA (2016). The many roles of glutamate in metabolism. J Ind Microbiol Biotechnol.

