# Supplementary figures and images for: The metabolic reprogramming of γ-aminobutyrate in oral squamous cell carcinoma
Source: BMC Oral Health. 2024 Apr 5;24:418. doi: 10.1186/s12903-024-04174-0 (PMC10996254; doi:10.1186/s12903-024-04174-0)

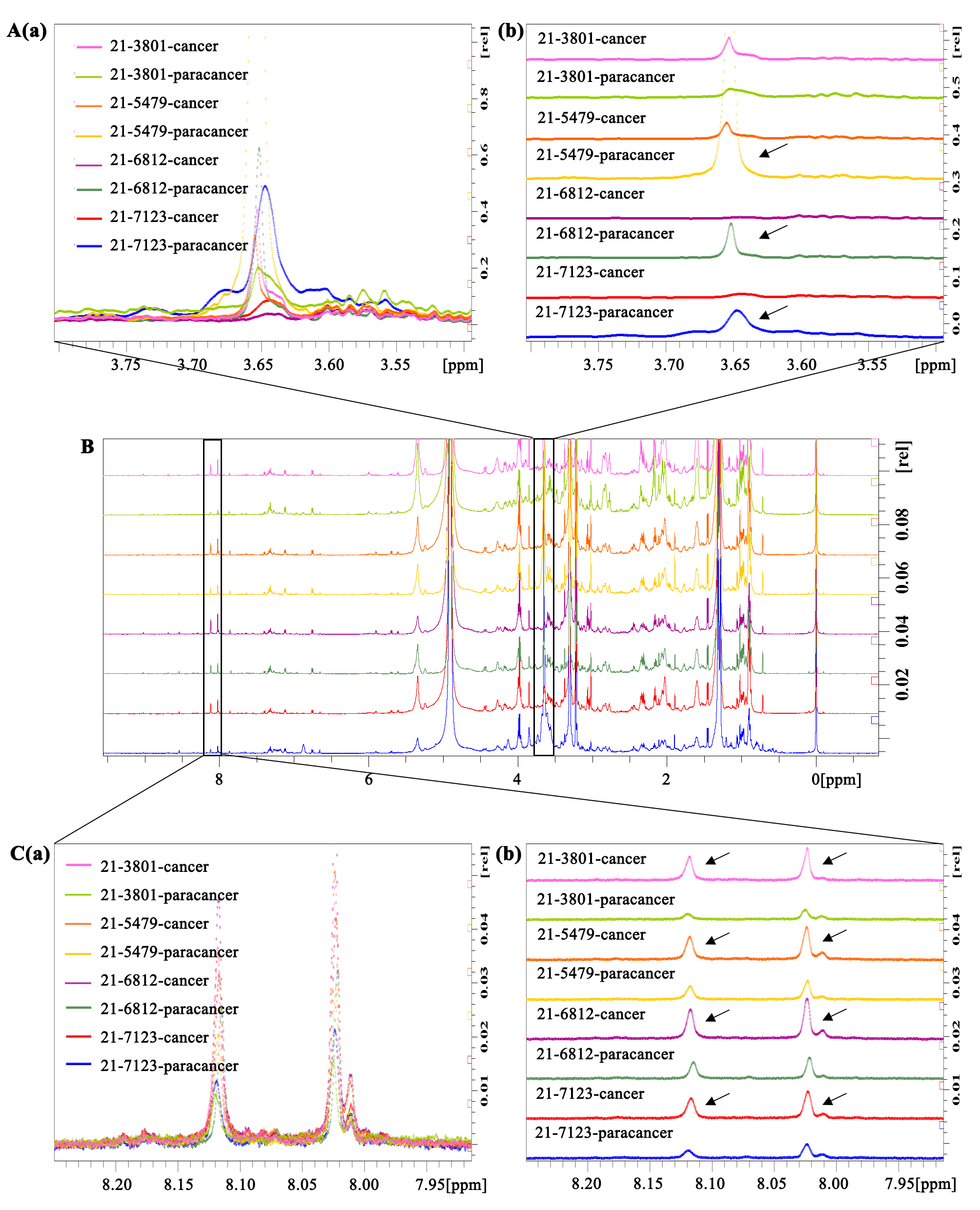

Supplement: Supplementary file 3 — Supplementary Material 3 [file 12903_2024_4174_MOESM3_ESM.jpg]

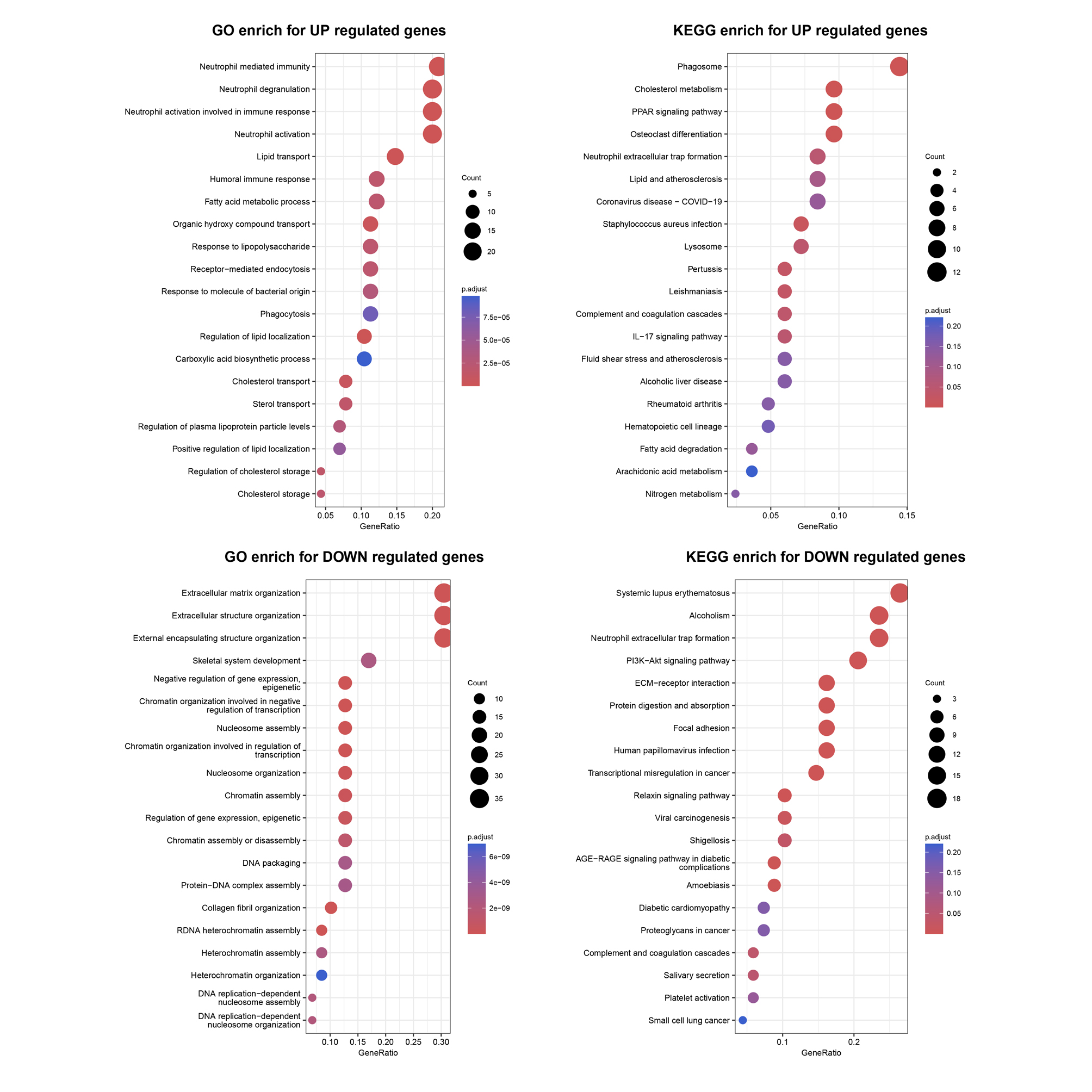

Supplement: Supplementary file 4 — Supplementary Material 4 [file 12903_2024_4174_MOESM4_ESM.jpg]

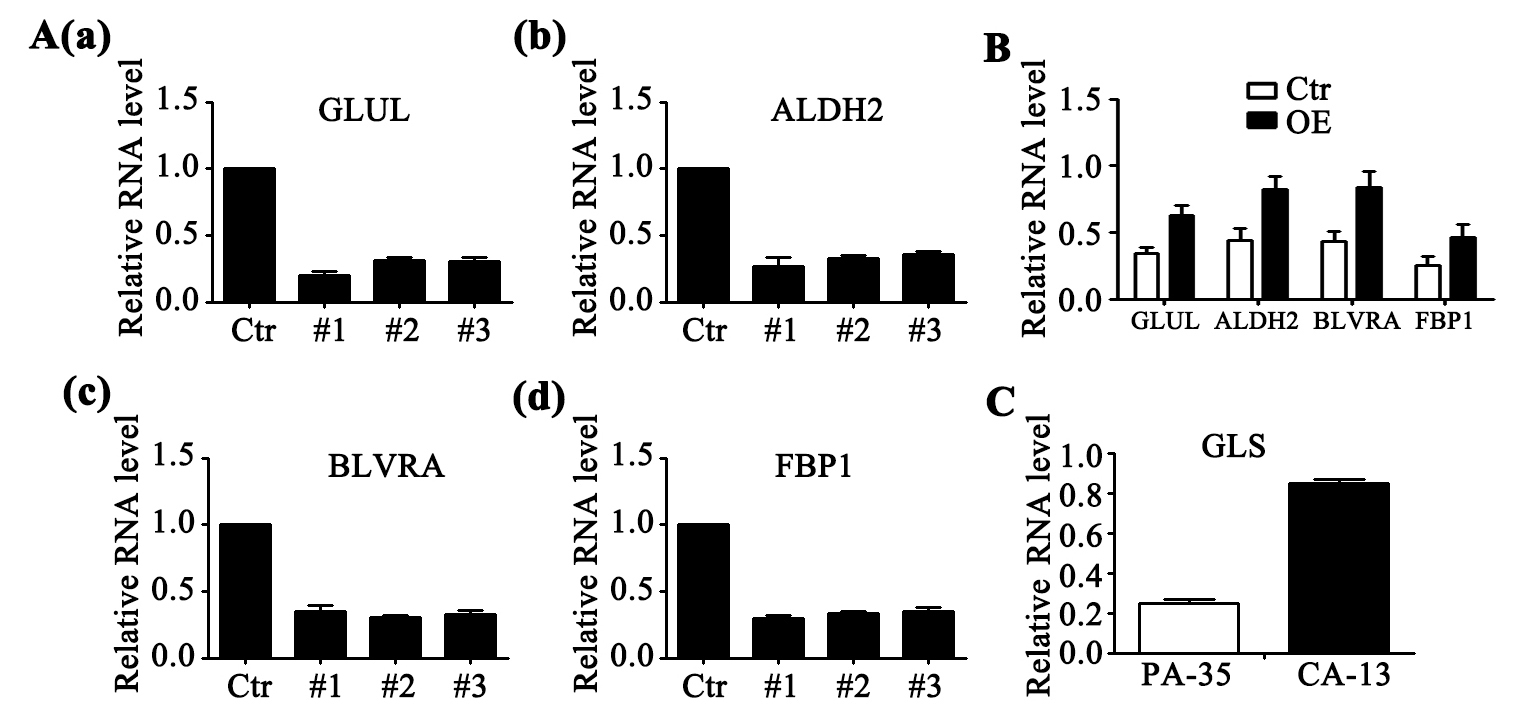

Supplement: Supplementary file 5 — Supplementary Material 5 [file 12903_2024_4174_MOESM5_ESM.jpg]

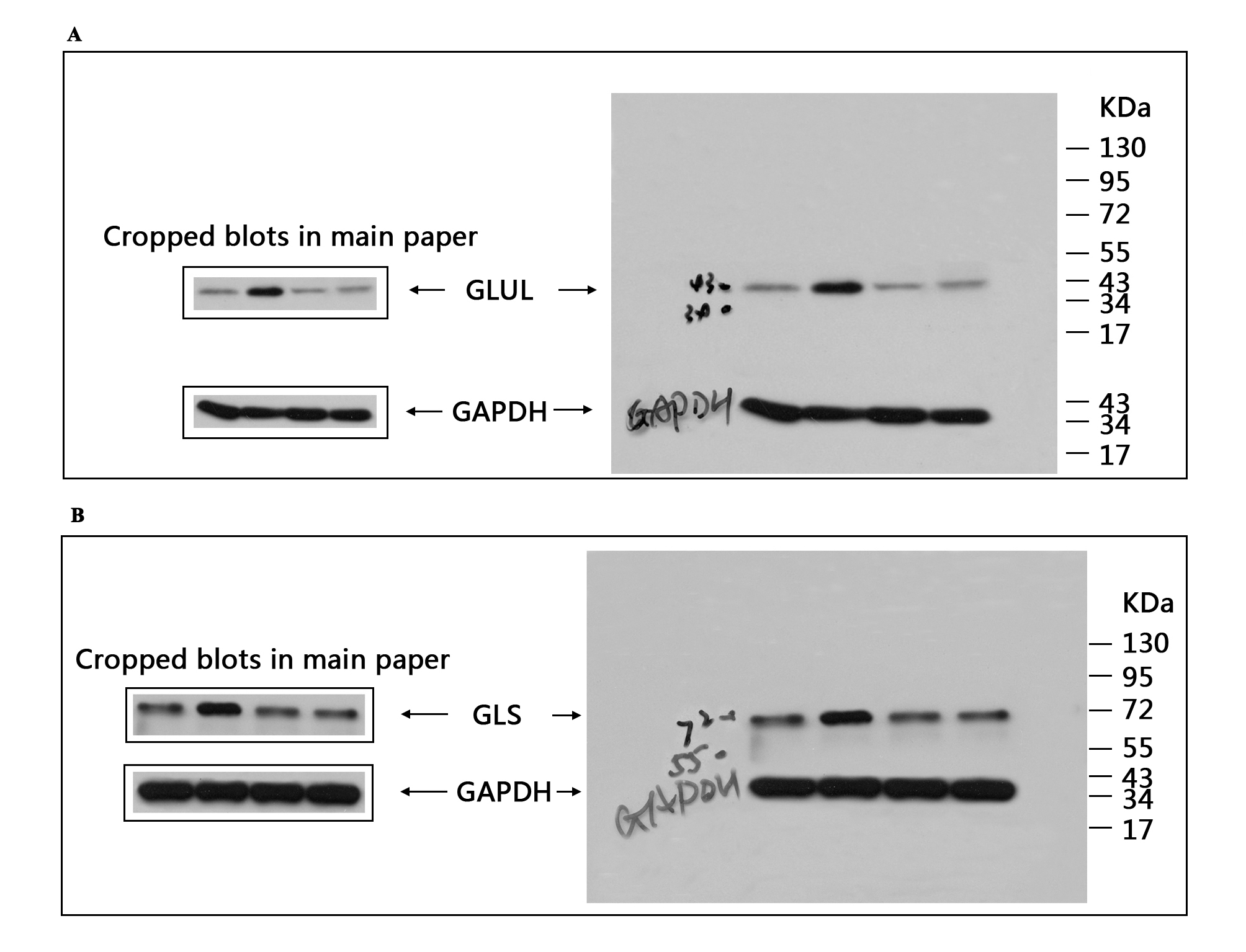

Supplement: Supplementary file 6 — Supplementary Material 6 [file 12903_2024_4174_MOESM6_ESM.jpg]

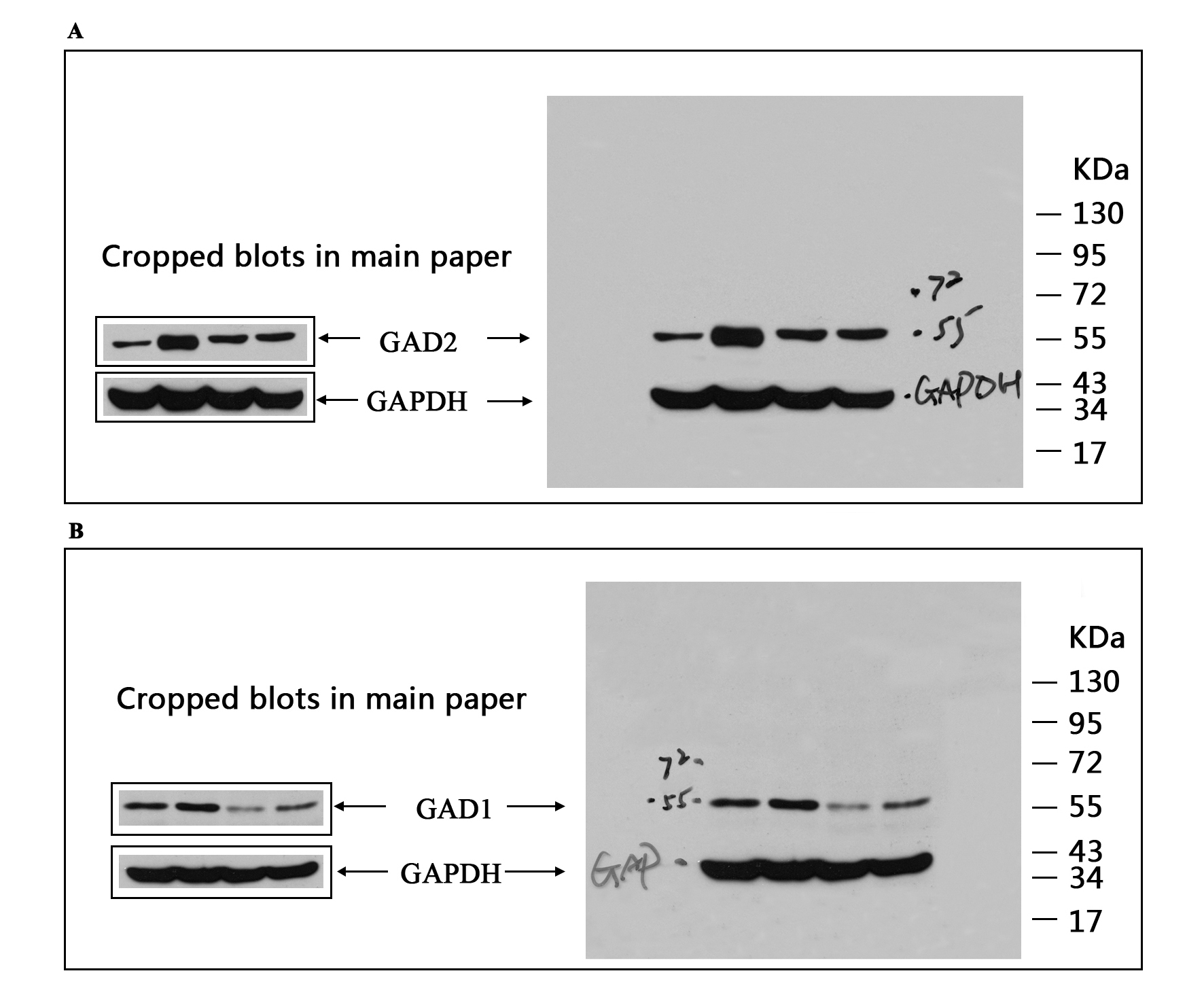

Supplement: Supplementary file 7 — Supplementary Material 7 [file 12903_2024_4174_MOESM7_ESM.jpg]
