# Supplementary material for: The metabolic reprogramming of γ-aminobutyrate in oral squamous cell carcinoma
Source: BMC Oral Health. 2024 Apr 5;24:418. doi: 10.1186/s12903-024-04174-0 (PMC10996254; doi:10.1186/s12903-024-04174-0)
Supplement: Supplementary file 8 — Supplementary Material 8 [file 12903_2024_4174_MOESM8_ESM.pdf]

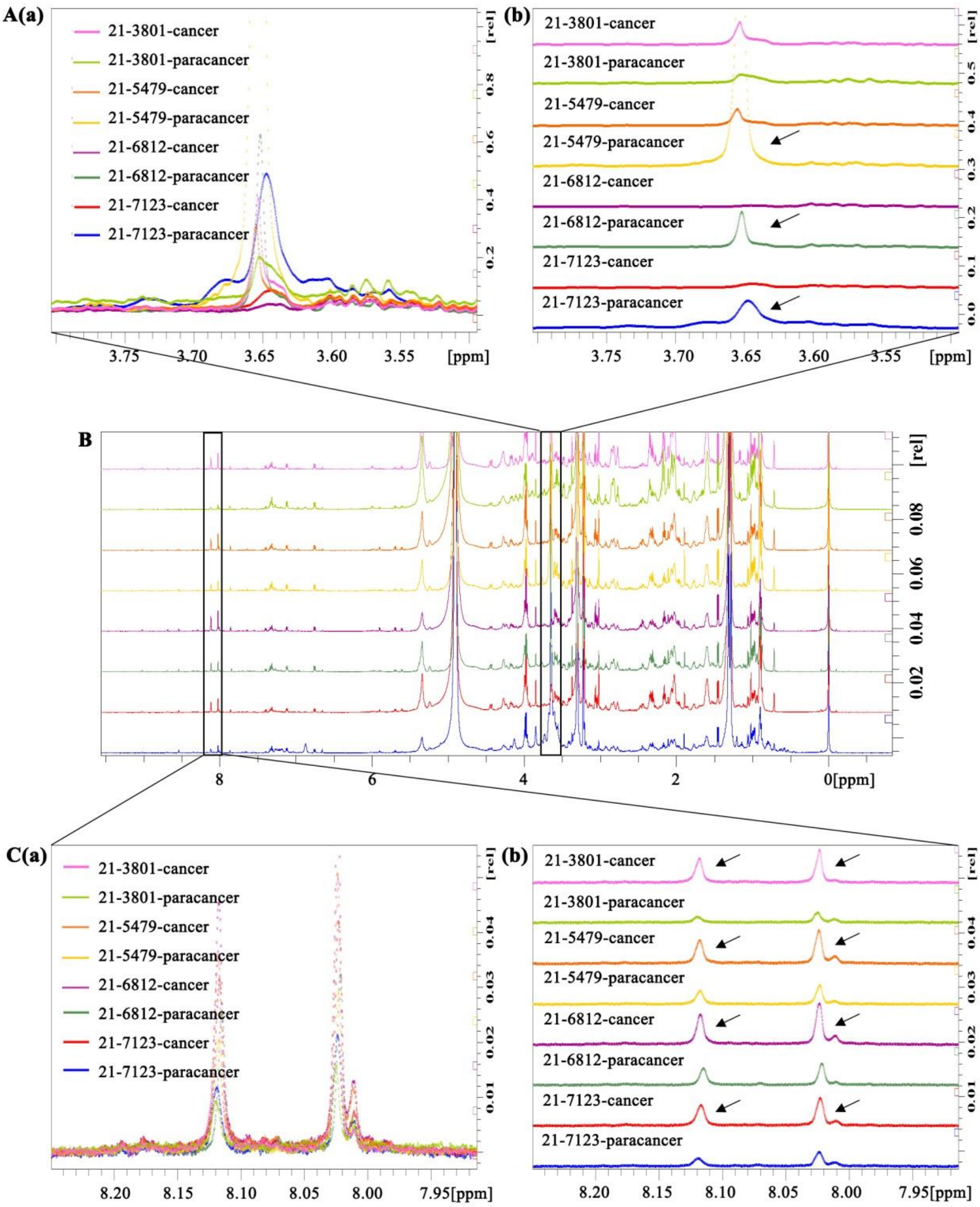

Supplementary Figure. 1 Analysis of metabolites in cancer and paracancerous tissues of OSCC patients.

The metabolites in cancer and paracancerous tissues of 4 OSCC patients (#21-3801, #21-5479, #21-6812 and #21-7123) were analyzed by ^{1}H-NMR. The relative chemical shift between 0-10 ppm was showed in **B.** The signal in the range of 3-4 ppm (**A**) and range of 7.90-8.25 ppm (**C**) were selected to export the original data. The layout was toggled as merged (a) or multiple displayed (b). The arrows show the sites of metabolite with density variation in cancer and paracancerous tissues.

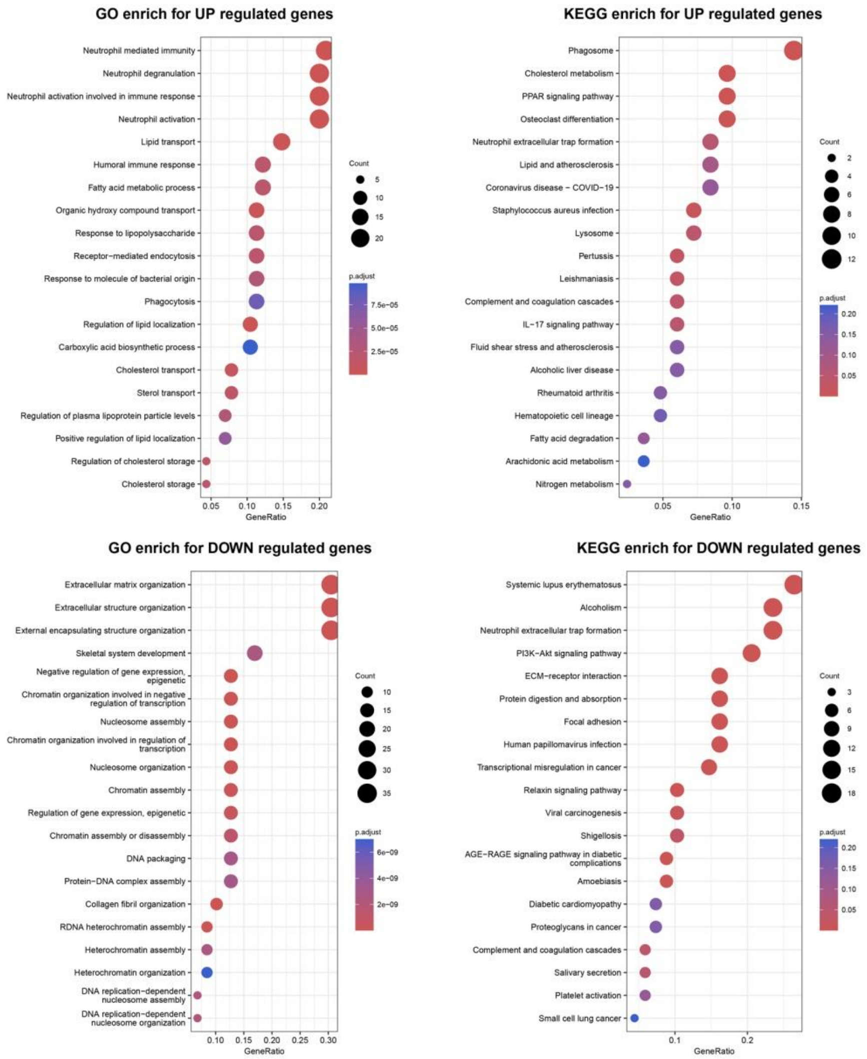

Supplementary Figure. 2 The gene expression in CA-13 and PA-35 cells.

CA-13 and PA-35 cells were sent for RNA-Seq. The gene expression in CA-13 cells was compared with that in PA-35 cells and analyzed by GO and KEGG enrichment.

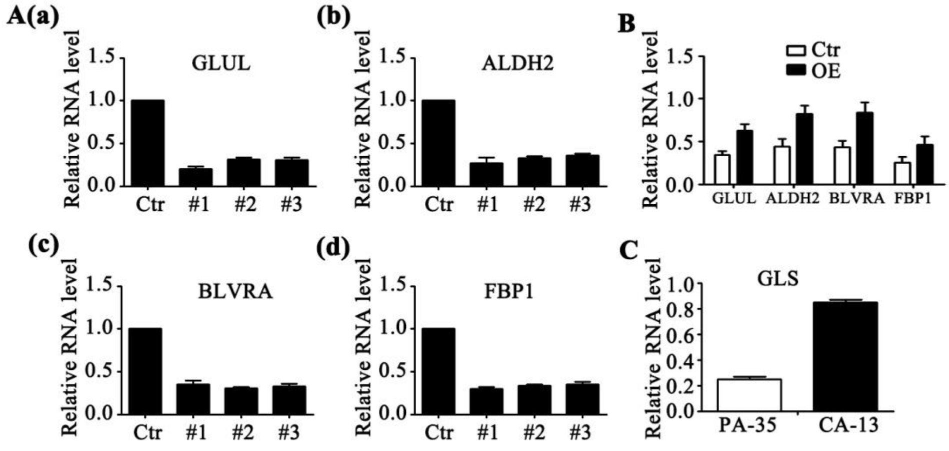

Supplementary Figure. 3 The RNA expression in indicated cells.

A. SiRNA controls (Ctr) or 3 siRNA oligos (#1,#2 and #3) of GLUL(a), ALDH2(b), BLVRA(c) or FBP1(d) genes were individually transfected into CA-13 cells. The RNA levels in siRNAs transfecting cells were measured by qRT-PCR and compared to siRNA control transfecting cells. **B**. The exogenous GLUL, ALDH2, BLVRA, FBP1 (OE) or their control (Ctr) plasmids were individually transfected into PA-35 cells. The RNA expression were dected by qRT-PCR. **C**. The RNA expression of GLS were dected by qRT-PCR in PA-35 and CA-13 cells.

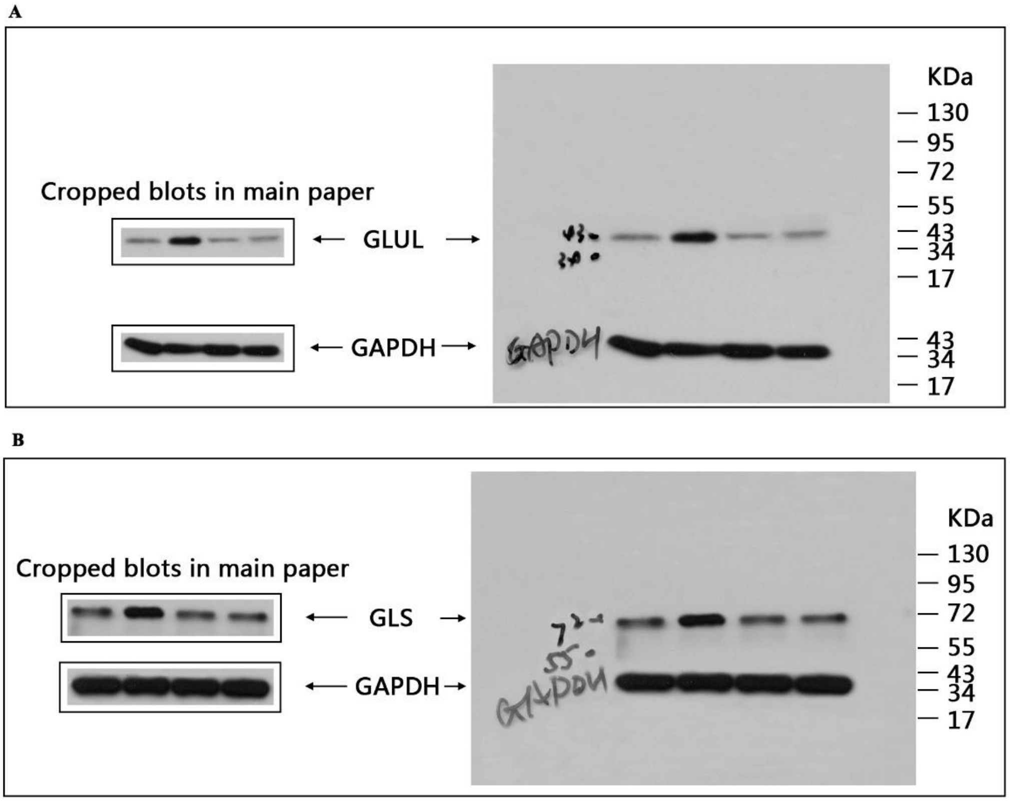

Supplementary Figure. 4 Supplementary original gel source data 1.

The original gel source data for fig 4D(a) and fig 4D(b) were provided as A and B.

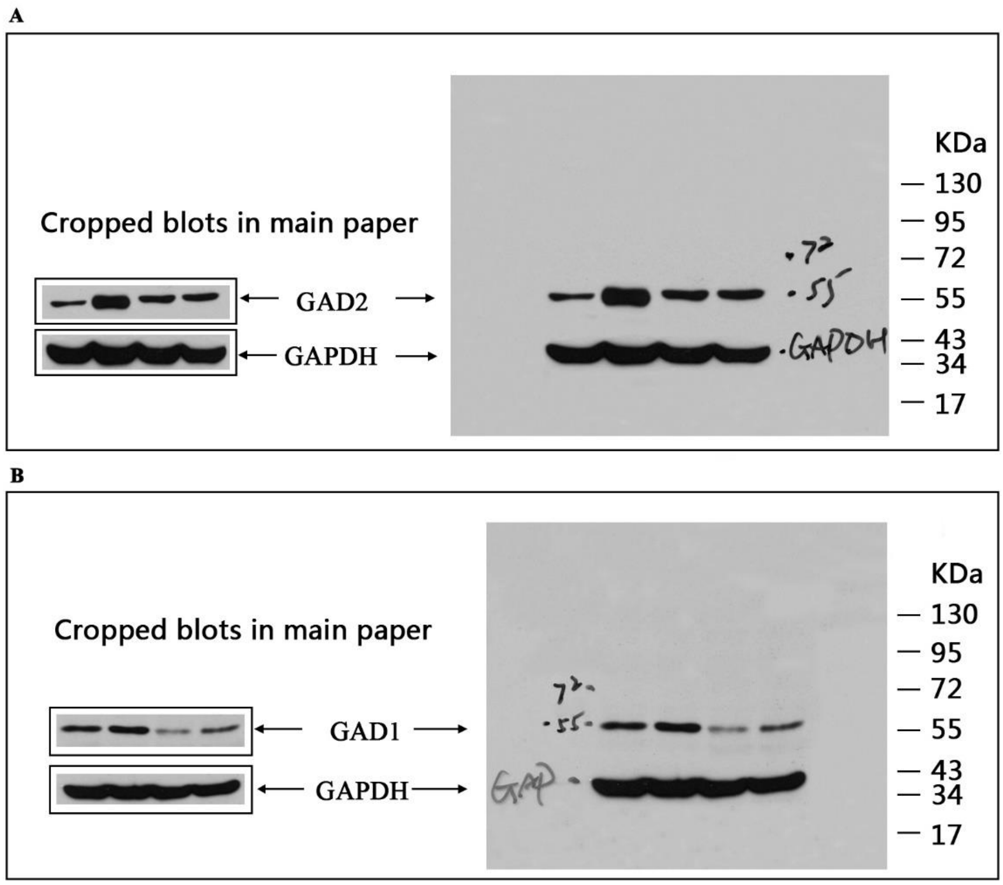

Supplementary Figure. 5 Supplementary original gel source data 2.

The original gel source data for fig 4D(c) and fig 4D(d) were provided as A and B.
